# Sex difference in the associations among risk factors with hepatitis B and C infections in a large Taiwanese population study

**DOI:** 10.3389/fpubh.2022.1068078

**Published:** 2022-11-30

**Authors:** Angela Chiunhsien Wang, Jiun-Hung Geng, Chih-Wen Wang, Da-Wei Wu, Szu-Chia Chen

**Affiliations:** ^1^Department of Post Baccalaureate Medicine, Kaohsiung Medical University, Kaohsiung, Taiwan; ^2^Department of Urology, Kaohsiung Municipal Siaogang Hospital, Kaohsiung Medical University, Kaohsiung, Taiwan; ^3^Department of Urology, Kaohsiung Medical University Hospital, Kaohsiung Medical University, Kaohsiung, Taiwan; ^4^Department of Internal Medicine, Kaohsiung Municipal Siaogang Hospital, Kaohsiung Medical University, Kaohsiung, Taiwan; ^5^Division of Hepatobiliary, Department of Internal Medicine, Kaohsiung Medical University Hospital, Kaohsiung Medical University, Kaohsiung, Taiwan; ^6^Research Center for Precision Environmental Medicine, Kaohsiung Medical University, Kaohsiung, Taiwan; ^7^Division of Pulmonary and Critical Care Medicine, Department of Internal Medicine, Kaohsiung Medical University Hospital, Kaohsiung Medical University, Kaohsiung, Taiwan; ^8^Division of Nephrology, Department of Internal Medicine, Kaohsiung Medical University Hospital, Kaohsiung Medical University, Kaohsiung, Taiwan; ^9^Faculty of Medicine, College of Medicine, Kaohsiung Medical University, Kaohsiung, Taiwan

**Keywords:** hepatitis B infections, hepatitis C infections, sex difference, Taiwan Biobank, risk factors

## Abstract

**Background:**

The prevalence rates of hepatitis B and C virus (HBV/HCV) infection are high in Taiwan, and both are common causes of chronic liver disease and its related complications. Therefore, the early detection of factors associated with HBV/HCV infection is important. The aim of this study was to explore these factors in a large cohort of Taiwanese participants in the Taiwan Biobank, and also to identify sex differences in these risk factors.

**Methods:**

It was an observational cohort study. The study enrolled 121,421 participants, and divided into four groups according to the presence or absence of HBV or HCV infection. Associations between risk factors with HBV or HCV infection were examined using multivariate logistic regression analysis.

**Results:**

The mean age of the 121,421 enrolled participants (43,636 men and 77,785 women) was 49.9 ± 11.0 years. The participants were stratified into four groups according to those with (*n* = 13,804; 11.4%) and without HBV infection (*n* = 107,617; 88.6%), and those with (*n* = 2,750; 2.3%) and without HCV infection (*n* = 118,671; 97.7%). Multivariable analysis revealed that male sex [vs. female sex; odds ratio [OR] = 1.346; 95% confidence interval (CI) = 1.282–1.414; *p* < 0.001] was significantly associated with HBV infection, whereas female sex (vs. male sex; OR = 0.642; 95% CI = 0.575–0.716; *p* < 0.001) was significantly associated with HCV infection. Furthermore, there were significant interactions between sex and age (*p* < 0.001), body mass index (*p* < 0.001), total cholesterol (*p* = 0.002), aspartate aminotransferase (*p* = 0.024), and estimated glomerular filtration rate (*p* = 0.012) on HBV infection. There were also significant interactions between sex and age (*p* < 0.001), hypertension (*p* = 0.010), fasting glucose (*p* = 0.031), and uric acid (*p* = 0.001) on HCV infection.

**Conclusion:**

In conclusion, sex differences were found among the risk factors for HBV and HCV infections in a large cohort of Taiwanese volunteers. When dealing with hepatitis B and hepatitis C, the physicians may need to pay attention to the differences between men and women to do different treatments.

## Introduction

The prevalence of hepatitis B virus (HBV) infection in Taiwan is 8.5%, and an estimated 1.85 million people are HBV carriers in Taiwan in 2019 (1). HBV can be transmitted during labor and delivery if the mother has had hepatitis shortly before and after delivery, and this accounts for the high prevalence of carriers in Taiwan (2). In addition, the prevalence of hepatitis C virus (HCV) is estimated to be around 1.6–2.5% in Taiwan (1). Most patients have a chronic HCV mono-infection, with an anti-HCV antibody positive rate as high as 30% in certain endemic areas located along the southwestern coast of Taiwan (3). The most common route of HCV transmission is parenteral, particularly through exposure to infected blood products, transplantation of infected tissues or organ grafts, and intravenous drug use (4). In areas where HBV infection is endemic, such as in Taiwan, 4.9% of HBV carriers are also positive for anti-HCV antibodies (5). HBV infection has been associated with 42% of all cases of cirrhosis and 56% of all cases of hepatocellular carcinoma (HCC) worldwide, compared to 21% and 20% for HCV infection (6, 7). In patients coinfected with both HBV and HCV who develop liver cirrhosis and HCC and other chronic liver diseases, the disease is usually more severe than in those with HCV or HBV mono-infection (8, 9). Therefore, early vaccination and treatment for HBV and HCV infections are of crucial importance.

Sex differences have been reported in diseases such as cardiovascular disease (10, 11). Women are more vulnerable than their male counterparts with respect to atypical symptoms due to differences in X and Y chromosomes, and postmenopausal decrease in estrogen levels (10, 11). Men tend to develop coronary artery diseases earlier than women with more severe atherosclerosis of coronary arteries due to the absence of sex hormone protection (10). In addition, female predominance has been reported in many autoimmune diseases along with sex differences in the clinical presentation, onset, progression and outcome (12, 13). Moreover, sex hormones have been reported to be a potential therapeutic option for some patients with autoimmune diseases (13). Sex differences have also been reported in HBV infection, with higher prevalence rates in males than in females worldwide (4) however, no sex differences have been reported in HCV infection (14). Most previous studies have emphasized the risk factors for HBV and HCV infections, however they have seldom focused on sex differences in these risk factors. Therefore, this study aimed to investigate sex differences in the correlations among risk factors for HBV and HCV infections in a large Taiwanese cohort of volunteers in the Taiwan Biobank (TWB).

## Materials and methods

### Study area

The Taiwan Ministry of Health and Welfare sponsored the TWB in response to the aging society with the aim of countering chronic diseases through the promotion of health.

### Study design

This study is an observational cohort study.

### Sample population and sample size

**Figure 1:**
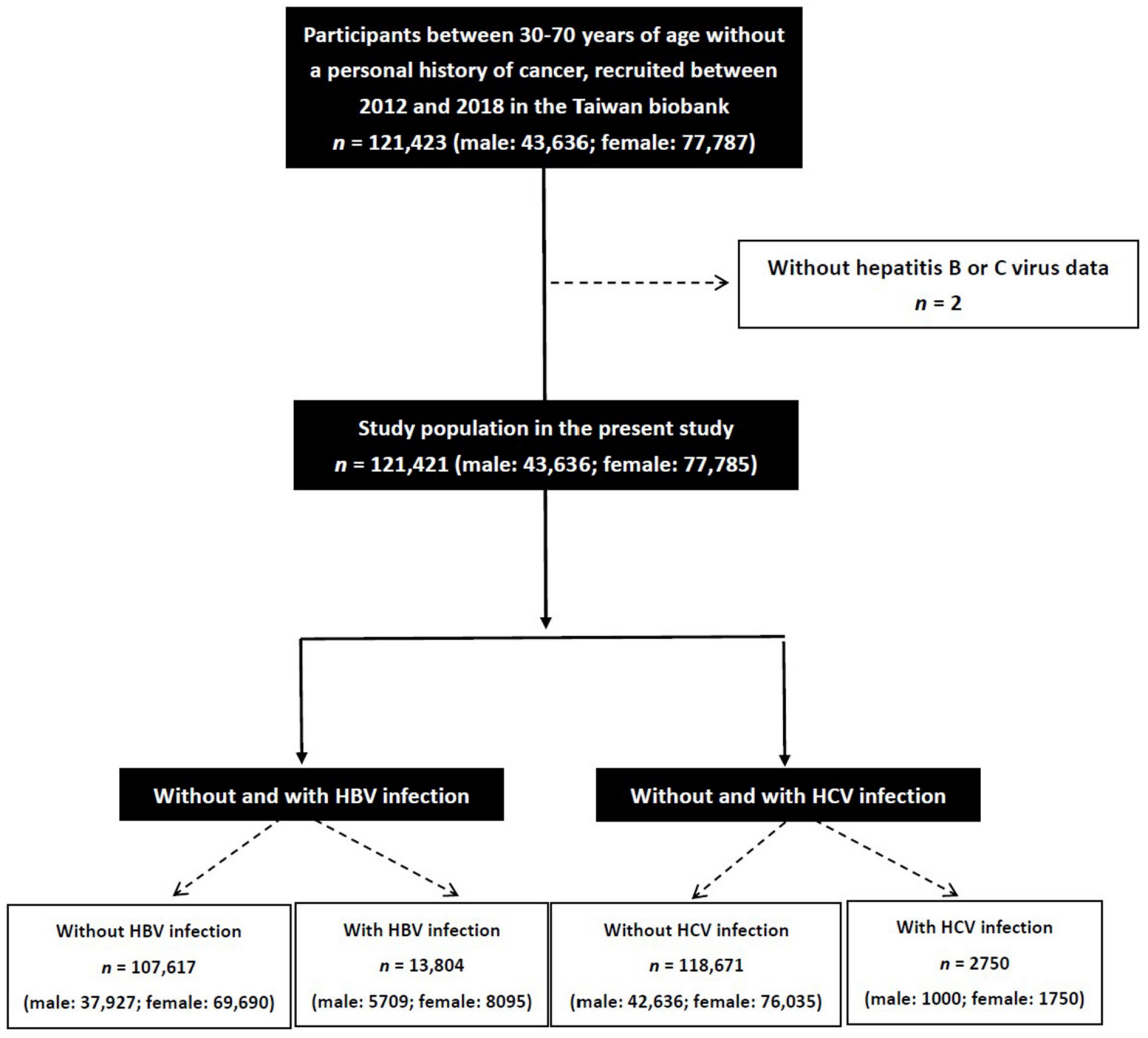
Flowchart of study population.

The study identified 121,423 participants in the TWB and excluded those with no data on HBV and HCV (*n* = 2). The remaining 121,421 participants (43,636 men and 77,785 women) were enrolled, and divided into four groups according to the presence or absence of HBV or HCV infection (Figure 1).

### Inclusion and exclusion criteria of TWB

All enrollees in the TWB are between 30 and 70 years of age and have no prior diagnosis of cancer. The database of the TWB contains information on the enrollees lifestyle habits, medical and genetic data (15, 16).

### Medical data, demographics, lifestyle habits, and laboratory data

The following variables were recorded: medical data, including the presence of hypertension and diabetes mellitus (DM); demographics, including sex and age; and lifestyle habits, including tobacco and alcohol use. Body mass index (BMI) was recorded as kg/m^2^. Fasting blood samples were obtained from all of the patients, and laboratory tests were conducted using an autoanalyzer (Roche Diagnostics GmbH, D-68298 Mannheim COBAS Integra 400). Laboratory data were also recorded at baseline after an 8-h fast including: total cholesterol, glucose, aspartate aminotransferase (AST), triglycerides, alanine aminotransferase (ALT), uric acid and estimated glomerular filtration rate (eGFR), which was estimated using the Modification of Diet in Renal Disease 4-variable equation (17). Serum levels of creatinine were calculated using the compensated Jaffé (kinetic alkaline picrate) method using a calibrator that could traced in isotope-dilution mass spectrometry (18). Hepatitis B surface antigen (HBsAg) and anti-HCV antibodies were tested using chemiluminescence (ADVIA Centaur, Siemens).

### Ethics statement

All of the participants signed informed consent forms, and the study was conducted according to the Declaration of Helsinki. The Institutional Review Board of Kaohsiung Medical University Hospital approved this study [KMUHIRB-E(I)-20210058]. Ethical approval for the TWB was granted by the Institutional Review Board on Biomedical Science Research, Academia Sinica, Taiwan and the Ethics and Governance Council of the TWB.

### Statistical analysis

Data are presented as number (%) or mean (±SD). For continuous variables, the independent *t-*test was used to analyze differences between groups, and the chi-square test was used for categorical variables. Associations between risk factors with HBV or HCV infection were examined using multivariate logistic regression analysis. An interaction *p* in logistic analysis was identified using the following formula: Model disease (y) = x1 + x2 + x1^*^x2 + covariates x1^*^x2, where y = HBV or HCV infection; x1 = sex; x2 = each risk factor; covariates = age, sex, DM, hypertension, alcohol and tobacco use, BMI, fasting glucose, triglycerides, total cholesterol, AST, ALT, eGFR and uric acid. Results were considered significant at *p* < 0.05. Statistical analysis was performed using SPSS for Windows (v26, SPSS Inc. Armonk, NY, USA).

## Results

The enrolled participants (*n* = 121,421; mean age 49.9 ± 11.0 years) were divided into four groups according to those with HBV infection (HBV+ group; *n* = 13,804; 11.4%) and without HBV infection (HBV– group; *n* = 107,617; 88.6%), and those with HCV infection (HCV+ group; *n* = 2750; 2.3%) and without HCV infection (HCV– group; *n* = 118,671; 97.7%).

### Differences in clinical characteristics among the four study groups

Compared to the HBV- group, the HBV+ group had a higher proportion of men, lower prevalence of hypertension and DM, more tobacco and alcohol use, lower eGFR. Lower levels of fasting glucose, triglycerides, total cholesterol, higher levels of ALT, and AST (Table 1). In addition, the HCV+ group were older, had a higher proportion of women, higher prevalence of hypertension and DM, more tobacco and alcohol use, higher levels of fasting glucose, ALT, AST, and uric acid, lower level of total cholesterol, and lower eGFR than the HCV- group (Table 1).

**Table 1:** Clinical characteristics of the study participants classified by the presence of HBV or HCV infections.

**Characteristics**	**All (*****n*** = **121,421)**			**All (*****n*** = **121,421)**		
	**HBV (−)**	**HBV (+)**	***p***	**HCV (−)**	**HCV (+)**	***p***
	**(*n* = 107,617)**	**(*n* = 13,804)**		**(*n* = 118,671)**	**(*n* = 2750)**	
Age (year)	49.9 ± 11.1	49.7 ± 9.9	0.083	49.8 ± 11.0	54.3 ± 9.7	<0.001
Male (%)	35.2	41.4	<0.001	35.9	36.3	<0.001
DM (%)	5.2	4.5	<0.001	5.1	7.9	<0.001
Hypertension (%)	12.4	11.2	<0.001	12.1	17.0	<0.001
Alcohol history (%)	8.4	9.1	0.005	8.4	13.2	<0.001
Smoking history (%)	27.0	29.1	<0.001	27.2	31.1	<0.001
Body mass index (kg/m^2^)	24.2 ± 3.8	24.2 ± 3.8	0.462	24.2 ± 3.8	24.3 ± 3.7	0.095
Laboratory parameters						
Fasting glucose (mg/dL)	96.0 ± 20.8	95.3 ± 19.9	<0.001	95.9 ± 20.6	98.6 ± 24.8	<0.001
Triglyceride (mg/dL)	117.0 ± 96.3	104.5 ± 73.5	<0.001	115.7 ± 93.8	113.6 ± 102.2	0.288
Total cholesterol (mg/dL)	196.3 ± 36.0	190.7 ± 34.0	<0.001	195.8 ± 35.8	187.8 ± 37.0	<0.001
AST (U/L)	24.6 ± 11.4	28.4 ± 19.2	<0.001	24.8 ± 12.1	32.9 ± 25.5	<0.001
ALT (U/L)	23.1 ± 18.9	29.4 ± 31.8	<0.001	23.6 ± 20.2	34.0 ± 40.3	<0.001
eGFR (mL/min/1.73 m^2^)	103.4 ± 24.0	102.3 ± 22.6	<0.001	103.3 ± 23.9	100.4 ± 24.1	<0.001
Uric acid (mg/dL)	5.4 ± 1.4	5.4 ± 1.4	0.306	5.4 ± 1.4	5.5 ± 1.4	<0.001

HBV, hepatitis B virus; HCV, hepatitis C virus; DM, diabetes mellitus; AST, aspartate aminotransferase; ALT, alanine aminotransferase; eGFR, estimated glomerular filtration rate.

### Determinants of HBV infection

The factors associated with HBV infection in all study participants (*n* = 121,421) in multivariable logistic regression analysis are shown in Table 2. After adjusting for age, sex, DM, hypertension, tobacco and alcohol use, BMI, fasting glucose, triglycerides, total cholesterol, ALT, AST, eGFR and uric acid, age (per 1 year; odds ratio [OR] = 1.002; *p* = 0.047), male (vs. female; OR = 1.346; *p* < 0.001), DM history (OR = 0.819; *p* < 0.001), hypertension history (OR = 0.856; *p* < 0.001), triglycerides (per 1 mg/dL; OR = 0.997; *p* < 0.001), total cholesterol (per 1 mg/dL; OR = 0.996; *p* < 0.001), AST (per 1 U/L; OR = 1.008; *p* < 0.001), ALT (per 1 U/L; OR = 1.009; *p* < 0.001), eGFR (per 1 mL/min/1.73 m^2^; OR = 0.997; *p* < 0.001), and uric acid (per 1 mg/dL; OR = 0.928; *p* < 0.001) were significantly associated with HBV infection.

**Table 2:** Determinants for HBV and HCV infections using multivariable logistic regression analysis.

**Variables**	**HBV**			**HCV**		
	**Multivariable**			**Multivariable**		
	**OR**	**95% CI**	***p***	**OR**	**95% CI**	***p***
Age (per 1 year)	1.002	1.000–1.004	0.047	1.044	1.039–1.048	<0.001
Male (vs. female)	1.346	1.282–1.414	<0.001	0.642	0.575–0.716	<0.001
DM	0.819	0.743–0.903	<0.001	0.885	0.750–1.045	0.151
Hypertension	0.856	0.805–0.910	<0.001	0.951	0.852–1.063	0.379
Alcohol history	1.027	0.960–1.098	0.438	1.532	1.351–1.737	<0.001
Smoking history	0.982	0.937–1.029	0.444	1.320	1.189–1.466	<0.001
Body mass index (per 1 kg/m^2^)	1.005	1.000–1.011	0.065	0.994	0.983–1.006	0.340
Fasting glucose (per 1 mg/dL)	0.999	0.998–1.000	0.075	1.002	1.000–1.004	0.029
Triglyceride (per 1 mg/dL)	0.997	0.997–0.998	<0.001	0.999	0.999–1.000	0.026
Total cholesterol (per 1 mg/dL)	0.996	0.996–0.997	<0.001	0.991	0.990–0.992	<0.001
AST (per 1 U/L)	1.008	1.006–1.010	<0.001	1.011	1.008–1.015	<0.001
ALT (per 1 U/L)	1.009	1.007–1.010	<0.001	1.004	1.002–1.006	<0.001
eGFR (per 1 mL/min/1.73 m^2^)	0.997	0.996–0.998	<0.001	0.999	0.998–1.001	0.500
Uric acid (per 1 mg/dL)	0.928	0.912–0.944	<0.001	1.054	1.018–1.091	0.003

Values expressed as odds ratio (OR) and 95% confidence interval (CI). HBV, hepatitis B virus; HCV, hepatitis C virus; DM, diabetes mellitus; AST, aspartate aminotransferase; ALT, alanine aminotransferase; eGFR, estimated glomerular filtration rate. Covariates in the multivariable model included age, sex, diabetes, hypertension, alcohol and smoking history, body mass index, fasting glucose, triglyceride, total cholesterol, AST, ALT, eGFR and uric acid.

### Determinants of HCV infection

The factors associated with HCV infection in all study participants (*n* = 121,421) in multivariable logistic regression analysis are also shown in Table 2. After multivariable analysis, age (per 1 year; OR = 1.044; *p* < 0.001), male (vs. female; OR = 0.642; *p* < 0.001), alcohol use (OR = 1.532; *p* < 0.001), tobacco use (OR = 1.320; *p* < 0.001), fasting glucose (per 1 mg/dL; OR = 1.002; *p* = 0.029), triglycerides (per 1 mg/dL; OR = 0.999; *p* = 0.026), total cholesterol (per 1 mg/dL; OR = 0.991; *p* < 0.001), AST (per 1 U/L; OR = 1.011; *p* < 0.001), ALT (per 1 U/L; OR = 1.004; *p* < 0.001), and uric acid (per 1 mg/dL; OR = 1.054; *p* = 0.003) were significantly associated with HCV infection.

### Determinants of HBV by sex

The factors associated with HBV infection by sex in multivariable logistic regression analysis are shown in Table 3. In the male participants (*n* = 43,636), DM history (OR = 0.772; *p* < 0.001), hypertension history (OR = 0.876; *p* = 0.002), BMI (per 1 kg/m^2^; OR = 0.990; *p* = 0.029), triglycerides (per 1 mg/dL; OR = 0.998; *p* < 0.001), total cholesterol (per 1 mg/dL; OR = 0.995; *p* < 0.001), AST (per 1 U/L; OR = 1.006; *p* < 0.001), ALT (per 1 U/L; OR = 1.010; *p* < 0.001), eGFR (per 1 mL/min/1.73 m^2^; OR = 0.998; *p* = 0.009), and uric acid (per 1 mg/dL; OR = 0.928; *p* < 0.001) were significantly associated with HBV infection. In the female participants (*n* = 77,785), age (per 1 year; OR = 1.004; *p* = 0.002), hypertension history (OR = 0.855; *p* < 0.001), BMI (per 1 kg/m^2^; OR = 1.015; *p* < 0.001), triglycerides (per 1 mg/dL; OR = 0.997; *p* < 0.001), total cholesterol (per 1 mg/dL; OR = 0.997; *p* < 0.001), AST (per 1 U/L; OR = 1.010; *p* < 0.001), ALT (per 1 U/L; OR = 1.008; *p* < 0.001), eGFR (per 1 mL/min/1.73 m^2^; OR = 0.997; *p* < 0.001), and uric acid (per 1 mg/dL; OR = 0.922; *p* < 0.001) were significantly associated with HBV infection.

**Table 3:** Determinants for HBV by the presence of different sex using multivariable logistic regression analysis.

**Variables**	**Male (*****n*** = **43,636)**			**Female (*****n*** = **77,785)**			
	**Multivariable**			**Multivariable**			
	**OR**	**95% CI**	***p***	**OR**	**95% CI**	***p***	**Interaction** ***p***
Age (per 1 year)	0.998	0.995–1.001	0.172	1.004	1.001–1.007	0.002	<0.001
DM	0.772	0.672–0.886	<0.001	0.872	0.760–1.001	0.052	0.162
Hypertension	0.876	0.804–0.954	0.002	0.855	0.783–0.933	<0.001	0.203
Alcohol history	1.025	0.950–1.107	0.522	1.053	0.913–1.214	0.481	0.845
Smoking history	1.013	0.955–1.075	0.661	0.957	0.883–1.037	0.280	0.269
Body mass index (per 1 kg/m^2^)	0.990	0.981–0.999	0.029	1.015	1.007–1.022	<0.001	<0.001
Fasting glucose (per 1 mg/dL)	0.999	0.998–1.001	0.461	0.998	0.997–1.000	0.067	0.492
Triglyceride (per 1 mg/dL)	0.998	0.997–0.998	<0.001	0.997	0.997–0.997	<0.001	0.741
Total cholesterol (per 1 mg/dL)	0.995	0.994–0.996	<0.001	0.997	0.996–0.998	<0.001	0.002
AST (per 1 U/L)	1.006	1.003–1.009	<0.001	1.010	1.006–1.014	<0.001	0.024
ALT (per 1 U/L)	1.010	1.008–1.012	<0.001	1.008	1.006–1.010	<0.001	0.157
eGFR (per 1 mL/min/1.73 m^2^)	0.998	0.996–0.999	0.009	0.997	0.996–0.998	<0.001	0.012
Uric acid (per 1 mg/dL)	0.928	0.906–0.950	<0.001	0.922	0.900–0.946	<0.001	0.239

Values expressed as odds ratio (OR) and 95% confidence interval. HBV, hepatitis B virus; HCV, hepatitis C virus; DM, diabetes mellitus; AST, aspartate aminotransferase; ALT, alanine aminotransferase; eGFR, estimated glomerular filtration rate. Covariates in the multivariable model included age, diabetes, hypertension, alcohol and smoking history, body mass index, fasting glucose, triglyceride, total cholesterol, AST, ALT, eGFR and uric acid.

### Interactions among risk factors and sex on HBV infection

There were significant interactions between sex and age (*p* < 0.001), BMI (*p* < 0.001), total cholesterol (*p* = 0.002), AST (*p* = 0.024), and eGFR (*p* = 0.012) on HBV infection (Table 3).

### Determinants of HCV by sex

The factors associated with HCV infection by sex in multivariable logistic regression analysis are shown in Table 4. In the male participants (*n* = 43,636), age (per 1 year; OR = 1.025; *p* < 0.001), alcohol use (OR = 1.616; *p* < 0.001), tobacco use (OR = 1.302; *p* < 0.001), fasting glucose (per 1 mg/dL; OR = 1.004; *p* < 0.001), triglycerides (per 1 mg/dL; OR = 0.999; *p* = 0.026), total cholesterol (per 1 mg/dL; OR = 0.990; *p* < 0.001), AST (per 1 U/L; OR = 1.008; *p* < 0.001), ALT (per 1 U/L; OR = 1.005; *p* < 0.001) were significantly associated with HCV infection. In the female participants (*n* = 77,785), age (per 1 year; OR = 1.055; *p* < 0.001), alcohol use (OR = 1.447; *p* = 0.004), tobacco use (OR = 1.473; *p* < 0.001), total cholesterol (per 1 mg/dL; OR = 0.991; *p* < 0.001), AST (per 1 U/L; OR = 1.016; *p* < 0.001), and uric acid (per 1 mg/dL; OR = 1.092; *p* < 0.001) were significantly associated with HCV infection.

**Table 4:** Determinants for HCV by the presence of different sex using multivariable logistic regression analysis.

**Variables**	**Male (*****n*** = **43,636)**			**Female (*****n*** = **77,785)**			
	**Multivariable**			**Multivariable**			
	**OR**	**95% CI**	***p***	**OR**	**95% CI**	***p***	**Interaction** ***p***
Age (per 1 year)	1.025	1.018–1.031	<0.001	1.055	1.049–1.061	<0.001	<0.001
DM	0.962	0.757–1.222	0.751	0.821	0.650–1.038	0.099	0.233
Hypertension	0.908	0.765–1.077	0.267	1.009	0.872–1.168	0.902	0.010
Alcohol history	1.616	1.394–1.872	<0.001	1.447	1.129–1.855	0.004	0.852
Smoking history	1.302	1.132–1.497	<0.001	1.473	1.259–1.724	<0.001	0.445
Body mass index (per 1 kg/m^2^)	0.982	0.963–1.002	0.084	0.997	0.983–1.012	0.687	0.151
Fasting glucose (per 1 mg/dL)	1.004	1.002–1.007	<0.001	0.999	0.996–1.002	0.506	0.031
Triglyceride (per 1 mg/dL)	0.999	0.998–1.000	0.026	1.000	0.999–1.000	0.293	0.127
Total cholesterol (per 1 mg/dL)	0.990	0.988–0.992	<0.001	0.991	0.990–0.992	<0.001	0.119
AST (per 1 U/L)	1.008	1.004–1.012	<0.001	1.016	1.010–1.021	<0.001	0.177
ALT (per 1 U/L)	1.005	1.003–1.008	<0.001	1.001	0.997–1.005	0.567	0.949
eGFR (per 1 mL/min/1.73 m^2^)	0.998	0.994–1.001	0.229	1.000	0.998–1.003	0.689	0.055
Uric acid (per 1 mg/dL)	0.997	0.948–1.049	0.908	1.092	1.041–1.145	<0.001	0.001

Values expressed as odds ratio (OR) and 95% confidence interval. HBV, hepatitis B virus; HCV, hepatitis C virus; DM, diabetes mellitus; AST, aspartate aminotransferase; ALT, alanine aminotransferase; eGFR, estimated glomerular filtration rate. Covariates in the multivariable model included age, diabetes, hypertension, alcohol and smoking history, body mass index, fasting glucose, triglyceride, total cholesterol, AST, ALT, eGFR and uric acid.

### Interactions among risk factors and sex on HCV infection

There were significant interactions between sex and age (*p* < 0.001), hypertension (*p* = 0.010), fasting glucose (*p* = 0.031), and uric acid (*p* = 0.001) on HCV infection (Table 4).

## Discussion

This study found that after adjusting for confounders, the male participants were significantly associated with HBV infection, whereas the female participants were significantly associated with HCV infection. Furthermore, the interactions between age, BMI, total cholesterol, AST, eGFR and sex on HBV infection were statistically significant, while the interactions between age, hypertension, fasting glucose, uric acid and sex on HCV infection were also statistically significant.

The important findings of this study include the significant associations between the male participants and HBV infection, and between the female participants and HCV. Males may be more prone to HBV infection due to the effect of sex hormones such as androgen and estrogen, which are produced in males and females, respectively (19). These hormones exert their biological function by binding to specific receptors, such as androgen receptors (ARs) or estrogen receptors (ERs), thereby activating signal transduction pathways (20, 21). The liver is considered to be a sexually dimorphic organ as it expresses both ARs and ERs, and thus it is responsive to sex hormones (22, 23). HBV X protein has been shown to enhance the transcriptional activity of ARs in an androgen concentration–dependent manner (24), which may amplify the sex difference in HBV-infected patients. In addition, a transgenic animal studies indicated that HBV replication and gene expression may be enhanced in the livers of males compared to females (25). It supported the idea of W4P mutation increasing the HBV virion replication and greater IL-6-mediated inflammation in male individuals (25). Another study demonstrated that NTCP, a functional receptor for HBV infection, especially in rs2296651 variant which is typically Asian-specific, was significantly associated with a decreased risk of HBV infection in Taiwanese women (26). These genetic and animal studies may partly explain the male predominance in HBV infection.

The finding of this study showed a significant association between HCV infection and the female participants in this study is consistent with several other studies (27, 28). However, several reviews (14, 29) have reported no sex disparity in HCV infection, and other studies (4, 30) have even reported that HCV infection is more common in men than in women. The major transmission route of HCV in Taiwan used to be through iatrogenic pathways. Nowadays, the risk of iatrogenic exposure has diminished, and HCV infection is sporadic (31, 32). A possible reason for the higher susceptibility to HCV in women may be because women have less access to medical support than men (33). In addition, since most HCV-infected patients are asymptomatic (34), they tend to have low awareness of the disease and therapeutic options. Therefore, HCV can incubate in these patients for a long time without being noticed. In addition, HCV-related HCC develops less frequently in women, with slower disease progression and better treatment response, resulting in higher overall survival (30). Taken together, these factors may explain the high prevalence of HCV infection in women.

Another finding of this study is that the interaction between BMI and sex on HBV infection was statistically significant, with a positive correlation in the women and negative correlation in the men. There is emerging evidence that sex disparity plays an important role in the distribution of adipose tissue (35). Men accumulate most of their fat in visceral adipose tissue, and women typically gain greater amounts subcutaneous (gluteal-femoral) fat (35). This is thought to be driven by hormone status and sex chromosome components. Many previous studies have reported that visceral adiposity triggers hepatic carcinogenesis, which may explain why HCC affects obese men more than obese women (36). However, another study (37) found that the association between BMI and HCC was U-shaped for men and linear for women, which is similar to the results of this study. Nevertheless, this report (37) did not differentiate whether HCC was HBV-related or not. Moreover, obesity is an inflammatory disease and also a risk factor for autoinflammatory and autoimmune diseases (35). Therefore, females are more affected by autoimmunity (12, 13). Another potential reason may be poor activation of the HBV vaccination in obese women, but increased seroprotection as they lose weight (38). However, there is currently no evidence of greater HBV vaccination activation in obese males than in obese females.

This study also found that the interaction between fasting glucose and sex on HCV infection was statistically significant, with an association between high fasting glucose and HCV infection in the male participants but not in the female participants. One review study (39) found that women had lower fasting plasma glucose in an oral glucose tolerance test (OGTT) than men. Gonadal hormones also have been implicated in this glucose homeostasis, as postmenopausal women receiving estrogen replacement therapy have been shown to have a lower level of fasting glucose and impaired glucose tolerance (39). In addition, insulin sensitivity is higher in women than in men, with women having a higher capacity for insulin secretion and incretin response than men (40). All of these factors are consistent with the high prevalence of DM in men. In recent years, numerous studies have focused on the association between HCV infection and glucose intolerance. It has been suggested that HCV infection could induce insulin resistance (41–43). Most studies (41, 44) have shown that HCV eradication decreases the risk of insulin resistance, while a few studies (45) have reported no association between HCV clearance and metabolic syndrome. Taken together, these findings support that HCV infection is associated with high fasting glucose, especially in men.

The significant interaction between uric acid and sex on HCV infection is also an important finding of this study. This study found that high uric acid was associated with HCV infection in the female participants but not in the male participants. Men have higher serum uric acid than women (46–48). Although glomerular filtration and urinary urate excretion are similar in both sexes, uric acid clearance and the fractional excretion of uric acid are significantly higher in women than in men (47). Sex hormones have also been suggested to play an important role in this process. In an animal study, estrogen level was shown to influence uric acid clearance by estradiol inhibiting the uric acid-generating enzyme xanthine oxidase isolated from rat livers (49). A recent study found that inhibition of the SLC2A9 genotype, which encodes a renal uric acid reuptake transporter, resulted in hypouricemia (48). The epidemiology of the SLC2A9 genotype was found to be about 1.2% in men and 6% in women (48). In addition, variation in the expression of SLC2A9 was also associated with estrogen level, as it was higher in premenopausal women and lower in postmenopausal women (48). HCV infection is associated with numerous metabolic diseases, including uric acid dysregulation. One Taiwanese study (50) indicated an inverse association between the severity of liver disease in HCV-infected males and serum uric acid. As mentioned, women with HCV infection are more likely to be older, which implies a low estrogen level and therefore an increased likelihood of hyperuricemia. These findings may be supported by the finding of this present study that the HCV-infected women had a higher serum uric acid level than the men.

The main strengths of this study include that it enrolled a large number of healthy community-dwelling participants, and comprehensive adjustment for confounding factors. However, there are also several limitations. First, certain medications may affect hypertension, fasting glucose and lipids, however the TWB does not contain information on medications. Second, this study could not determine the duration of HBV and HCV infection due to the cross-sectional nature of the study. Consequently, it was not possible to elucidate the causal relationship between medication use and HCV infection. Further longitudinal studies are warranted to investigate sex differences and incident HBV and HCV infection. Third, the genotypes of HBV and HCV and the severity the infections could not be ascertained. Fourth, as all of the enrolled participants were of Han ethnicity, the generalizability of the findings may be limited.

## Conclusion

In conclusion, this study demonstrated sex differences between HBV and HCV infections. The male participants were significantly associated with HBV infection, whereas the female participants were significantly associated with HCV infection. Further, there were sex differences in the associations among the risk factors with HBV and HCV infection in this study of a large population of community-dwelling Taiwanese participants. When dealing with hepatitis B and hepatitis C, the physicians may need to pay attention to the differences between men and women to do different treatments.

## Data availability statement

The data analyzed in this study is subject to the following licenses/restrictions: the data underlying this study is from the Taiwan Biobank. Due to restrictions placed on the data by the Personal Information Protection Act of Taiwan, the minimal data set cannot be made publicly available. Data may be available upon request to interested researchers. Requests to access these datasets should be directed to S-CC, scarchenone@yahoo.com.tw.

## Ethics statement

The studies involving human participants were reviewed and approved by the Institutional Review Board of Kaohsiung Medical University Hospital approved this study [KMUHIRB-E(I)-20210058]. The patients/participants provided their written informed consent to participate in this study.

## Author contributions

S-CC: conceptualization, methodology, validation, formal analysis, writing—review and editing, visualization, supervision, resources, project administration, and funding acquisition. J-HG, C-WW, D-WW, and S-CC: software and investigation. ACW and S-CC: data curation. ACW: writing—original draft preparation. All authors contributed to the article and approved the submitted version.

## Funding

This work was supported partially by the Research Center for Precision Environmental Medicine, Kaohsiung Medical University, Kaohsiung, Taiwan from The Featured Areas Research Center Program within the framework of the Higher Education Sprout Project by the Ministry of Education (MOE) in Taiwan and by the Kaohsiung Medical University Research Center Grant (KMU-TC111A01 and KMUTC111IFSP01).

## Conflict of interest

The authors declare that the research was conducted in the absence of any commercial or financial relationships that could be construed as a potential conflict of interest.

## Publisher's note

All claims expressed in this article are solely those of the authors and do not necessarily represent those of their affiliated organizations, or those of the publisher, the editors and the reviewers. Any product that may be evaluated in this article, or claim that may be made by its manufacturer, is not guaranteed or endorsed by the publisher.
